# Altered DNA methylation in children born to mothers with rheumatoid arthritis during pregnancy

**DOI:** 10.1136/annrheumdis-2018-214930

**Published:** 2019-05-29

**Authors:** Hilal Ince-Askan, Pooja R Mandaviya, Janine F Felix, Liesbeth Duijts, Joyce B van Meurs, Johanna M W Hazes, Radboud J E M Dolhain

**Affiliations:** 1 Department of Rheumatology, Erasmus MC, University Medical Center Rotterdam, Rotterdam, The Netherlands; 2 Department of Internal Medicine, Erasmus MC, University Medical Center Rotterdam, Rotterdam, The Netherlands; 3 The Generation R Study Group, Erasmus MC, University Medical Center Rotterdam, Rotterdam, The Netherlands; 4 Department of Epidemiology, Erasmus MC, University Medical Center Rotterdam, Rotterdam, The Netherlands; 5 Department of Pediatrics, Erasmus MC, University Medical Center Rotterdam, Rotterdam, The Netherlands; 6 Department of Pediatrics, Division of Respiratory Medicine and Allergology, Erasmus MC, University Medical Center Rotterdam, Rotterdam, The Netherlands; 7 Department of Pediatrics, Division of Neonatology, Erasmus MC, University Medical Center Rotterdam, Rotterdam, The Netherlands

**Keywords:** pregnancy, rheumatoid arthritis, dna methylation, epigenetics

## Abstract

**Objectives:**

The main objective of this study was to determine whether the DNA methylation profile of children born to mothers with rheumatoid arthritis (RA) is different from that of children born to mothers from the general population. In addition, we aimed to determine whether any differences in methylation are associated with maternal RA disease activity or medication use during pregnancy.

**Methods:**

For this study, genome-wide DNA methylation was measured at cytosine-phosphate-guanine (CpG) sites, using the Infinium Illumina HumanMethylation 450K BeadChip, in 80 blood samples from children (mean age=6.8 years) born to mothers with RA. As controls, blood samples from 354 children (mean age=6.0 years) from the population-based Generation R Study were used. Linear mixed models were performed to investigate differential methylation between the groups, corrected for relevant confounders.

**Results:**

A total of 147 CpGs were differentially methylated between blood samples of children born to mothers with RA and the control blood samples. The five most significantly associated CpGs were cg06642177, cg08867893, cg06778273, cg07786668 and cg20116574. The differences in methylation were not associated with maternal RA disease activity or medication use during pregnancy.

**Conclusions:**

DNA methylation at 147 CpGs differed between children born to mothers with RA and children born to mothers from the general population. It remains unknown whether the identified associations are causal, and if so whether they are caused by the disease or treatment. More research, including replication of these results, is necessary in order to strengthen the relevance of our findings for the later-life health of children born to mothers with RA.

**Key messages**

**What is already known about this subject?**

- Adverse exposures in early life are associated with later-life health.
- Epigenetic changes are thought to be one of the underlying mechanisms.
- There is not much known about the consequences of maternal rheumatoid arthritis (RA) on the offsprings’ long-term health.

**What does this study add?**

- DNA methylation is different in children born to mothers with RA compared with mothers from the general population.

**How might this impact on clinical practice or future developments?**

- Maternal RA disease during pregnancy might have lifelong consequences for the offspring.
- More research in this particular field must be undertaken.

Adverse exposures in early life are associated with later-life health, which is referred to as the developmental origins of health and disease hypothesis.^1–5^ Epigenetic processes are thought to be one of the mechanisms underlying the associations of early-life exposures and later-life health outcomes.^6 7^ DNA methylation is the best studied and understood epigenetic modification.^8 9^ Factors that have been demonstrated to be associated with fetal DNA methylation include maternal disease,^7^ malnutrition,^10–13^ smoking,^14^ placental insufficiency,^15^ corticosteroids,^16^ folate depletion^17^ and cytokines.^18^ DNA methylation usually occurs at cytosine-phosphate-guanine (CpG) sites.^8 9^ The effect of hypermethylation and hypomethylation on gene expression depends on the CpG location.^19 20^ The most pronounced changes in DNA methylation occur during early pregnancy.^7 11^

During embryogenesis, there are three germ layers that form in the developing fetus (ectoderm, mesoderm and endoderm). When DNA methylation is altered in early pregnancy, all germ layers are affected.^21 22^

Rheumatoid arthritis (RA) may be considered as an adverse exposure during pregnancy.^23^ Therefore, it is plausible that maternal RA may induce changes in fetal DNA methylation, and that it is related with the later-life health of the offspring. Interleukin-6 is known to influence DNA methylation.^18^ RA treatment during pregnancy includes among others sulfasalazine (SSZ) and corticosteroids such as prednisone. SSZ is a known folate antagonist that crosses the placenta and could influence DNA methylation in this respect.^17 24^ Furthermore, corticosteroids might influence DNA methylation.^16^ Especially during early pregnancy, when the placenta is not completely developed, prednisone passively diffuses to the fetus.^25–28^

In the current study we investigated whether the DNA methylation profile of children born to mothers with RA was different from that of children born to mothers from the general population. Furthermore, we investigated whether any differentially methylated CpGs were associated with RA disease activity or medication use during pregnancy, or with indicators of future metabolic and cardiovascular diseases. In addition, we examined whether these CpGs were associated with the expression of genes using expression quantitative trait methylation (eQTM) analysis.

## Methods

### Study population

#### FEPRA study

This study is embedded in the Pregnancy-induced Amelioration of Rheumatoid Arthritis (PARA) study, a prospective cohort study on pregnancy and RA.^29^ From 2002 to 2008, 369 female patients with RA who had a wish to conceive (or already pregnant) were enrolled.^30 31^ After participation in the PARA study, 196 children and their parents were invited to participate in a follow-up study, the FEtal Programming in Rheumatoid Arthritis (FEPRA) study. For this study, 108 children with a mean age of 6.8 years (SD=1.3) visited Erasmus Medical Centre in Rotterdam, and the parents of 85 children (all of European ancestry) gave informed consent for studies on DNA methylation of their children. Furthermore, the parents of 71 children provided cheek swabs from their children. There were no statistical differences in baseline characteristics between the participating and non-participating group.

#### Generation R Study

The control group consisted of children with a mean age of 6.0 years (SD=0.4), included in the Generation R Study, a population-based prospective cohort study from pregnancy onwards in Rotterdam, the Netherlands.^32^ In this study, all pregnant women living in Rotterdam with a delivery date between April 2002 and January 2006 were invited to participate, and 9778 mothers were enrolled.^32^ At the age of 6 years, DNA methylation was measured in a subgroup of 493 children of European ancestry.

### Data collection

#### FEPRA study

In the PARA study, data on mother (eg, disease activity (with the Disease Activity Score in 28 joints using C reactive protein levels, DAS28-CRP^3^)) and child were collected.^31^ For the FEPRA study, data on blood pressure, growth and body composition of the children were measured. Also, blood, which is a mesoderm germ layer derivate, was drawn for DNA methylation analysis.^33^ Cheek swabs were collected for the analysis of DNA methylation in buccal epithelial cells, which is an ectoderm germ layer derivate.

#### Generation R Study

In the Generation R Study, mothers were seen three times during pregnancy. The children were followed from birth until childhood. Data collection in children and their mothers included questionnaires, detailed physical examinations and blood sampling.^32^

### DNA methylation analysis

Genomic DNA was extracted from whole peripheral blood samples and from the cheek swab samples. Bisulfite conversion of 500 ng of genomic DNA was performed using the Zymo EZ-96 DNA Methylation Kit (Zymo Research, Irvine, California, USA) according to the manufacturer’s protocol.

Genomic methylation profiling was performed using the Infinium Illumina HumanMethylation 450K BeadChip arrays (Illumina, San Diego, USA) according to the manufacturer’s protocol. The Illumina array measures methylation status of 485 512 CpG sites in the gene and non-gene regions across the entire human genome. To prevent possible batch effects, blood and cheek swab samples were measured in the same run.

#### Quality control and normalisation

The data were preprocessed using the minfi package in R V.3.4.1 (www.r-project.org). Samples with incomplete or poor bisulfite conversion, extension, hybridisation or specificity were excluded.^34^ In addition, samples with sex mismatch and samples with a call rate <95% were removed. This quality control (QC) was done separately for blood samples and for cheek swab samples. During QC, 5 blood and 14 cheek swab samples from the FEPRA study were excluded, resulting in 80 and 57 samples, respectively. From the Generation R blood samples, 27 were excluded due to corticosteroid use or RA disease in the mother, and 32 were excluded during QC, resulting in 441 blood samples. In addition, 87 cases with missing data from the Generation R Study were excluded, leaving 354 samples to analyse. The intensity values were then quantile normalised using the incorporating Control Probe Adjustment and reduction of global CORrelation (CPACOR) workflow.^34^ Methylation at each CpG was calculated as the beta value (beta=intensity of the methylated allele (M)/(intensity of the unmethylated allele (U)+M+100)), containing values from 0 to 1. Blood cell composition of the samples was estimated using the Houseman method with the Reinius reference set.^35 36^ The Reinius reference set is not yet validated in children. However, it is the best method available, and it has been used in other epigenetic studies in children.^37 38^ Probes with single nucleotide polymorphisms (SNPs) at single base extension, probes with improper binding, and CpGs on the X and Y chromosome were removed from the analysis.^37–40^ From the initial 485 512 CpGs, 32 057 were excluded during QC, leaving 453 456 CpGs for analysis.

### Statistical analysis

For all subjects, descriptive statistics were calculated using Stata V.14.1 (https://www.stata.com/stata14/). Student’s t-tests and χ^2^ tests were used to compare the baseline characteristics. For these analyses, p values <0.05 were considered statistically significant.

Linear mixed models were performed to analyse differential methylation between the groups, using R. The first model was created to compare the blood samples from the FEPRA study with the blood samples of the Generation R Study to determine whether the DNA methylation profile of children born to mothers with RA was different from that of children born to mothers from the general population. This model was corrected for biological covariates (age, body mass index (BMI) SD scores (SDS), adjusted for age and sex according to the Dutch reference values, using the Growth Analyser (V.4.0; Growth Analyser, Rotterdam, the Netherlands, www.growthanalyser.org,) sex, gestational age at delivery, maternal age, folic acid use during pregnancy, socioeconomic status (SES), maternal smoking and white blood cell subtypes), technical covariates (technical batch effects (array identifier (ID) and position on array)) and five hidden confounders. Technical covariates were added as random effects in the models. The hidden confounders were calculated using the *CATE* package^41 42^ while correcting for the group (RA vs non-RA offsprings), all biological covariates and technical covariates. This resulted in hidden confounders that were independent of all included covariates. The *BACON* package^42^ was used to correct for unobserved covariates in order to reduce test statistic bias and inflation. The genomic inflation factor (λ)^43^ was calculated. After QC, 453 456 CpGs remained for analysis. Therefore, a Bonferroni-adjusted p value of 0.05/453 456=1.10×10^−^^7^ was used.^44^

CpGs were annotated for nearby genes with the Genomic Regions Enrichment of Annotations Tool,^45^ a general method to present epigenetic data.^37 38 40^ Significant CpGs from the first analysis were then further analysed within the blood samples of the FEPRA study to explore if RA disease activity, prednisone use or SSZ use during pregnancy would explain the differences in methylation found in the first model. After that, two linear mixed models were created with the significant CpGs to identify whether these were associated with the BMI SDS or the fat percentage SDS of the child, as indicators of future metabolic disease. These models were analysed in the 80 blood samples of the FEPRA study. CpGs with a p value below 0.05/147=3.4×10^−4^ were considered statistically significant.

The significant CpGs found in the first analysis were also analysed in DNA derived from buccal epithelial cells, obtained by cheek swabs from the FEPRA study, to explore if the differentially methylated CpGs were also differentially methylated in that germ layer derivate, as a kind of validation of the results. For this analysis, CpGs with a p value below significance level of 3.4×10^−4^ were considered statistically significant.

### eQTM analysis

eQTMs are sites at which DNA methylation is known to influence the expression of one or more genes.^46^ To analyse whether any of the significant CpGs were linked to the expression of nearby genes, an eQTM analysis was performed. For these analyses the online BIOS QTL browser (https://molgenis26.target.rug.nl/downloads/biosqtlbrowser/) was used.^39^

## Results

### Participants

**Figure 1:**
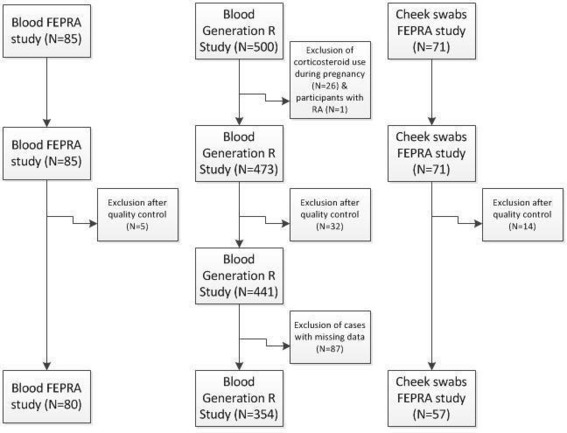
Flow chart of the study population and exclusion of participants. FEPRA, FEtal Programming in Rheumatoid Arthritis; RA, rheumatoid arthritis.

The flow chart of the study population is shown in figure 1. A total of 80 blood and 57 cheek swab samples from the FEPRA study (children born to mothers with RA) and 354 blood samples from the Generation R Study (children born to mothers from the general population) remained for analysis.

Descriptive statistics of the study population are presented in table 1. Overall, children from the FEPRA study were slightly older compared with children in the Generation R Study (p<0.001). In the FEPRA study, 45% of the women did not start using folic acid before or in early pregnancy compared with 92.7% in the Generation R Study (p<0.001). Approximately half of the women (47.5%) from the FEPRA study had a high SES compared with 68.4% of the women from the Generation R Study (p<0.001). One woman (1.3%) from the FEPRA study and 86 women (24.3%) from the Generation R Study smoked periconceptionally or at any time during pregnancy (p<0.001).

**Table 1:** Descriptive statistics of study population

	FEPRA study (n=80)	Generation R Study (n=354)
Age child* (years), mean (SD)	6.8 (1.3)†	6.0 (0.4)†
BMI SDS child,* mean (SD)	−0.14 (0.87)†	0.18 (0.74)†
Sex of the child		
Male, n (%)	46 (57.5)	176 (49.7)
Female, n (%)	34 (42.5)	178 (50.3)
Maternal age at delivery (years), mean (SD)	32.9 (3.9)	32.5 (4.0)
Gestational age (weeks), mean (SD)	39.5 (2.0)†	40.2 (1.5)†
Gestational age <37 weeks, n (%)	8 (10.0)†	6 (1.7)†
Folic acid		
Start before pregnancy, n (%)	25 (31.3)†	212 (59.9)†
Start in early pregnancy, n (%)	19 (23.8)†	116 (32.8)†
No use, n (%)	36 (45.0)†	26 (7.3)†
SES based on educational level		
Low, n (%)	9 (11.3)†	4 (1.1)†
Middle, n (%)	33 (41.3)†	108 (30.5)†
High, n (%)	38 (47.5)†	242 (68.4)†
Maternal smoking,‡ n (%)	1 (1.3)†	86 (24.3)†
DAS28-CRP(3) third trimester, mean (SD)	3.3 (1.1)	–
Use of medication ≥1 trimester		
Only prednisone use, n (%)	17 (21.3)	–
Only sulfasalazine use, n (%)	14 (17.5)	–
Combination, n (%)	13 (16.3)	–
Prednisone dose (mg), median (IQR)		
First trimester	7.5 (2.5–10.0)	–
Second trimester	7.5 (5.0–10.0)	–
Third trimester	6.3 (5.0–10.0)	–
No medication use, n (%)	36 (45.0)	–
Fat percentage SDS, mean (SD)	0.24 (0.97)	–

*At time of the blood sampling.

†P<0.001.

‡During pregnancy.

BMI, body mass index; DAS28-CRP(3), Disease Activity Score in 28 joints using C reactive protein levels; FEPRA, FEtal Programming in Rheumatoid Arthritis; SDS, SD score; SES, socioeconomic status.

### DNA methylation analysis

**Figure 2:**
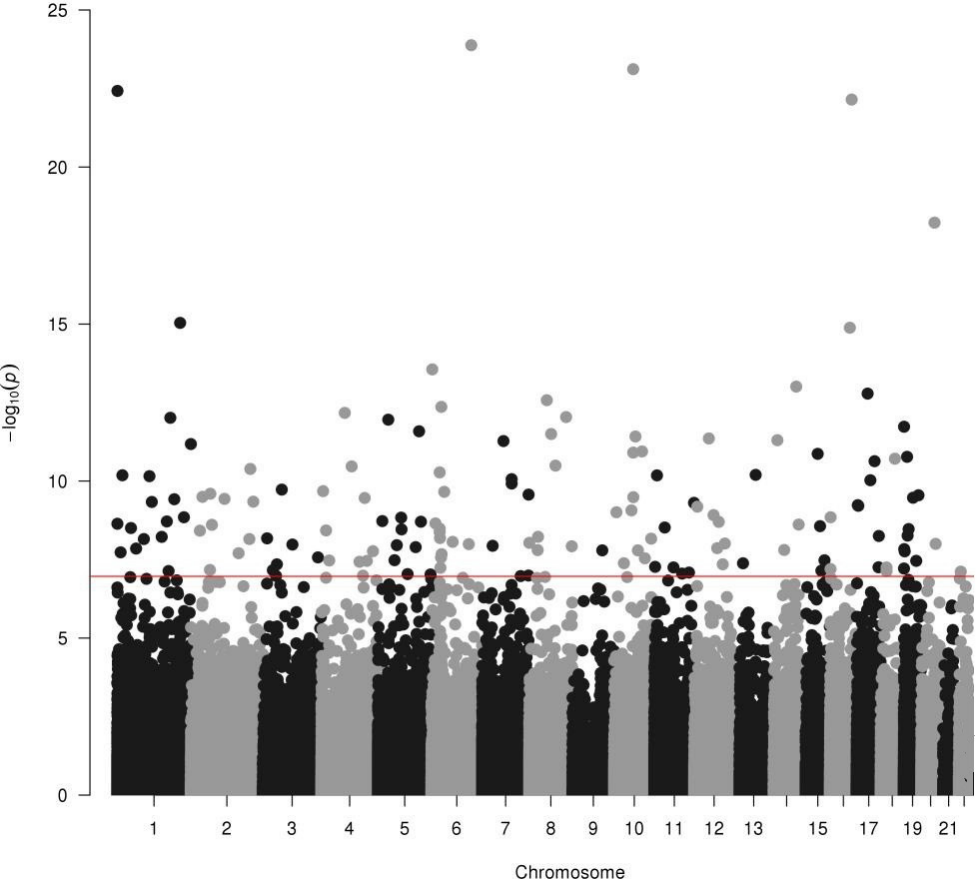
Results of the analysis of DNA methylation in children born to mother with rheumatoid arthritis (FEPRA study) compared with children born to women from the general population (Generation R Study). The chromosomes are depicted on the x-axis, and the −log p value on the y-axis. The red line represents the Bonferroni threshold for a significance of p=1.10×10^−^^7^. FEPRA, FEtal Programming in Rheumatoid Arthritis.

**Figure 3:**
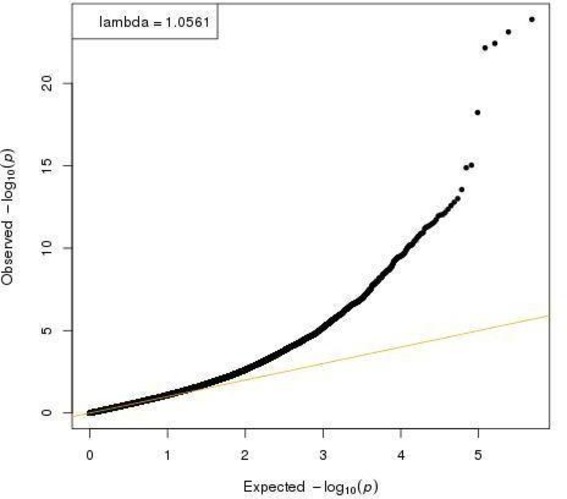
QQ plot of observed versus expected p values from the analysis of DNA methylation in children born to mother with rheumatoid arthritis (FEPRA study) compared with children born to women from the general population (Generation R Study). FEPRA, FEtal Programming in Rheumatoid Arthritis.

In the first linear mixed model, blood samples from the FEPRA study were compared with blood samples from the Generation R Study, corrected for the covariates mentioned in the Methods section. In total, 147 CpGs were significantly differentially methylated between children in the FEPRA study and children in the Generation R Study (figure 2). The QQ plot is shown in figure 3. The genomic inflation factor (λ) was 1.06.

In table 2 the five most significant CpGs and the five CpGs with the largest effect sizes (within the significant CpGs) are described in detail. The complete list of significantly associated CpGs is provided as an online supplementary file. A positive beta represents higher methylation levels in children born to mothers with RA. Methylation was higher in children born to mothers with RA than in those from the general population at all five CpGs with the lowest p values. Methylation at the five CpGs with the largest effect sizes was lower in children born to mothers with RA, with the exception of cg06656994.

10.1136/annrheumdis-2018-214930.supp1 Supplementary data

**Table 2:** The five most significant CpGs (white rows) and the five CpGs with the largest effect size (grey rows) from the linear mixed model: DNA methylation in blood samples from children born to mothers with RA (FEPRA study) compared with children born to mothers from the general population (Generation R Study)

CpG	Beta*	SE	P value	Nearest gene (±bp)	Chr	bp	Location†
cg06642177	0.028	0.002	1.32×10^−24^	*SLC2A12* (−122 529)	6	134 496 341	–
cg08867893	0.018	0.002	7.66×10^−24^	*ZNF365* (+221)	10	64 134 160	–
cg06778273	0.024	0.002	3.77×10^−23^	*TNFRSF18* (+4995)	1	1 137 117	–
cg07786668	0.026	0.002	7.11×10^−23^	*ZFHX3* (−10 142)	16	73 092 391	–
cg20116574	0.019	0.002	5.91×10^−19^	*NCOA5* (+435)	20	44 718 168	Promoter
cg16930947	−0.050	0.008	3.22×10^−11^	–	8	88 984 447	–
cg01485645	−0.044	0.006	1.64×10^−13^	*MLLT6* (+303)	17	36 862 199	Promoter
cg12360123	−0.043	0.008	1.61×10^−08^	–	10	79 984 532	Enhancer
cg06656994	0.038	0.005	9.67×10^−13^	*FAM163A* (+903)	1	179 713 176	Enhancer
cg17483482	−0.037	0.006	4.62×10^−10^	–	1	117 152 162	–

*Beta represents the difference in DNA methylation at the given CpG site in children born to mothers with RA (FEPRA study) as compared with children born to mothers from the general population (Generation R Study).

†Location in promoter, enhancer or unknown (–).

bp, base pair; Chr, chromosome; CpG, cytosine-phosphate-guanine; FEPRA, FEtal Programming in Rheumatoid Arthritis; RA, rheumatoid arthritis.

### Subsequent analysis of DNA methylation

The mean DAS28-CRP(3) in the first, second and third trimesters (3.6, 3.5 and 3.3, respectively) were highly correlated (>0.6). The DAS28-CRP(3) in the third trimester was available in all patients. Therefore, this timepoint was chosen for the analysis. None of the 147 CpGs were significantly associated with maternal RA disease activity (DAS28-CRP(3)) in the third trimester or medication (prednisone or SSZ) use. In addition, none of the CpGs were associated with BMI SDS or fat percentage SDS in the children.

### Analysis in buccal epithelial cells

A total of 10 out of the 147 CpGs significantly associated with maternal RA in blood were also associated in buccal epithelial cells. From these, four were in the same direction as in blood (table 3). CpG cg11336323 was located in a promoter region.

**Table 3:** CpGs that were differentially methylated in the same direction in both blood and in buccal epithelial cells

CpG	Beta*	SE	P value	Nearest gene (±bp)	Chr	bp	Location†
cg22998206	0.1029	0.022	4.40×10^−06^	–	12	49 239 429	–
cg03654106	0.0727	0.016	9.50×10^−06^	–	19	49 539 527	–
cg02613964	−0.058	0.014	7.57×10^−05^	–	3	44 690 321	–
cg11336323	−0.092	0.024	1.63×10^−04^	–	19	41 946 040	Promoter

*Beta represents the difference in DNA methylation at the given CpG site in buccal epithelial cells from children born to mothers with RA (FEPRA study) as compared with blood samples from children born to mothers from the general population (Generation R Study).

†Location in promoter, gene, enhancer or unknown (–).

bp, base pair; Chr, chromosome; CpG, cytosine-phosphate-guanine; FEPRA, FEtal Programming in Rheumatoid Arthritis; RA, rheumatoid arthritis.

### eQTM analysis

Two CpGs, cg21384971 and cg11220663, were associated with expression of the *COPZ2* and *ADD2* genes, respectively (table 4). These two genes were also the nearest genes to those CpGs.^45^ Both CpGs were hypermethylated in the children born to mothers with RA and were associated with decreased expression of COPZ2 and ADD2 in the BIOS eQTM lookup browser.

**Table 4:** Results from the eQTM analysis using the 147 CpGs significantly different in children born to mothers with RA (FEPRA study)

CpG	Beta*	SE*	P value*	Nearest genes	Beta GN†	SE GN†	P value GN†	Genes GN†
cg21384971	0.029	0.004	3.86×10^−13^	*COPZ2*	−0.073	0.039	1.79×10^−06^	COPZ2
cg11220663	0.023	0.003	1.68×10^−11^	*ADD2*	−0.121	0.039	3.32×10^−06^	ADD2

The positive betas in column ‘Beta’ represent hypermethylation, while the negative betas in column ‘Beta GN’ represent decreased gene expression.

*The columns beta, SE, p value and nearest genes represent the results from the analysis of DNA methylation in children born to mothers with RA (FEPRA study) as compared with children born to mothers from the general population (Generation R Study).

†The columns beta GN, SE GN, p value GN and genes GN represent the results from the BIOS eQTM lookup browser.

CpG, cytosine-phosphate-guanine; eQTM, expression quantitative trait methylation; FEPRA, FEtal Programming in Rheumatoid Arthritis; GN, GeneNetwork; RA, rheumatoid arthritis.

## Discussion

This is the first study investigating the differences in DNA methylation of children born to mothers with RA compared with children born to mothers from the general population. In this unique study, all participants were followed prospectively from pregnancy onwards. Our study showed differential DNA methylation between the two groups. The differentially methylated CpG sites were not associated with disease activity and/or medication use, nor to BMI SDS and fat percentage SDS.

In total, 147 CpGs were significantly associated with maternal RA after adjustment for multiple biological and technical covariates and hidden confounders. Of the five most significant CpGs, interestingly, two (cg06642177 and cg07786668) have been associated with myocardial infarction.^47^ The most significant CpG, cg06642177, is located on chromosome 6 near the *SLC2A12* gene,^48^ associated with insulin sensitivity,^48 49^ heart failure and diabetes^50^ in animal models. cg07786668, located on chromosome 16, is located in the *ZFHX3* gene. *ZFHX3* has been associated in multiple human studies with atrial fibrillation,^51–55^ coronary heart disease^56^ and obesity in a Korean population.^57^ cg20116574 was annotated to the *NCOA5*, a protein coding gene, which has been associated with diabetes mellitus type 2 in animal models.^58 59^

From the remaining 142 CpGs, 1 (cg17218495), annotated to the *SMARCA4* gene, has independently and significantly been associated with myocardial infarction.^47^ The other significant CpGs from our study have not been associated with disease phenotypes.

Out of the initial 147 significant CpGs, 10 were also significantly differentially methylated in buccal epithelial cells obtained by cheek swabs. From these, four were in the same direction as in blood. When DNA methylation is altered in more than one germ layer derivate, it is likely that these alterations occurred in early development.^21 22^ These four CpGs have not been associated with disease phenotypes in humans. Unfortunately, in the Generation R Study, DNA methylation in buccal epithelial cells was not available. There were no publicly available epigenetic data sets on buccal epithelial cells in healthy children with a similar age and background.

As mentioned before, CpGs that are associated with eQTMs can influence the expression levels of genes.^46^ In our study, from the 147 significant CpGs, 2 were present in the BIOS QTL browser. cg21384971 is associated with less expression of the *COPZ2* gene, which has been studied as a therapeutic opportunity for proliferation-independent selective killing of tumour cells.^60^ cg11220663 is associated with less expression of the *ADD2* gene, also known as beta-adducin. *ADD2* is involved in multiple pathogenic processes with a wide range of diseases.^61^ ADD2 gene variants are associated with hypertension,^62^ cancer^61^ and systemic lupus erythematosus.^63^

Pathway analysis of the genes annotated to the 147 significant CpGs, using the WebGestalt (WEB-based Gene Set AnaLysis Toolkit),^64^ did not result in significant pathways (data not shown).

Thus, some of the associated CpGs (cg06642177, cg07786668 and cg17218495) have been associated with cardiovascular disease in previous studies, while others are located in or near genes that are associated with cardiovascular or metabolic disease. Maternal RA during pregnancy, a chronic inflammatory disease, might be associated with later-life health and disease risk in the offspring.

None of the significant CpGs were associated with RA disease activity or medication use during pregnancy. However, this may have been due to a lack of power, since these analyses were performed in the 80 samples of the children born to women with RA. The same power problem also applied to the analysis of the CpGs with indicators for future metabolic and cardiovascular disease (BMI SDS and fat percentage).

Remarkably, a large percentage of women with RA did not use folic acid before or during pregnancy. Even though this is outside the scope of our study, this requires additional attention during preconceptional counselling.

Our study has some limitations. First, although in its kind it is a large study, a study on DNA methylation including 80 subjects and 354 controls is still relatively small. Despite this, a large number of CpGs reached significance. Correcting for biological and technical covariates, as well as hidden confounders, and using *BACON* resulted in a λ near 1, reflecting that there was no inflation. Second, we were not able to collect a new independent cohort of children born to mothers with RA to replicate the results. At the time our study was performed, there were no other comparable prospective studies available. Currently, European research groups are conducting new prospective cohort studies on the impact of RA on pregnancy and offspring. We encourage these research groups, possibly with international collaborations, to replicate our study.

Thus, the results of this study may support follow-up research of children born to mothers with RA. Based on our data, we recommend that at least indicators for future cardiovascular and metabolic disease should be considered. The effects of RA disease activity and medication use on DNA methylation should be investigated in studies with larger sample sizes. Furthermore, in the last years the use of tumour necrosis factor (TNF) inhibitors during pregnancy in patients with RA has increased. This often results in lower RA disease activity during pregnancy. Future research should also cover the effects of the use of TNF inhibitors on the differentially methylated CpGs in children born to mothers with RA.

In addition, it would be interesting for a future study to measure DNA methylation in the mothers of the children from our study and compare that with the methylation of their offspring. Since mothers and children live in a shared environment (generally), there may be an overlap in differentially methylated CpGs.

In conclusion, maternal RA during pregnancy is associated with differential DNA methylation in offspring. It remains unknown whether the identified associations are causal, and if so whether they are caused by the disease or treatment. Some of the differentially methylated CpGs or their nearby genes were associated with cardiovascular or metabolic disease. Maternal RA disease might have lifelong consequences for the offspring. However, more research in this particular field must be undertaken in order to strengthen the relevance of our findings.

## References

[1] Barouki R, Gluckman PD, Grandjean P, et al Developmental origins of non-communicable disease: implications for research and public health. Environ Health 2012;11 10.1186/1476-069X-11-42 PMC3384466 22715989

[2] Barker DJP Fetal origins of cardiovascular disease. Ann Med 1999;31:3–6. 10.1080/07853890.1999.11904392 28850284

[3] Ong KK, Dunger DB Perinatal growth failure: the road to obesity, insulin resistance and cardiovascular disease in adults. Best Pract Res Clin Endocrinol Metab 2002;16:191–207. 10.1053/beem.2002.0195 12064888

[4] Painter RC, de Rooij SR, Bossuyt PM, et al Early onset of coronary artery disease after prenatal exposure to the Dutch famine. Am J Clin Nutr 2006;84:322–7. 10.1093/ajcn/84.2.322 16895878

[5] Stein AD, Kahn HS, Rundle A, et al Anthropometric measures in middle age after exposure to famine during gestation: evidence from the Dutch famine. Am J Clin Nutr 2007;85:869–76. 10.1093/ajcn/85.3.869 17344511

[6] Turan N, Ghalwash MF, Katari S, et al DNA methylation differences at growth related genes correlate with birth weight: a molecular signature linked to developmental origins of adult disease? BMC Med Genomics 2012;5 10.1186/1755-8794-5-10 PMC3359247 22498030

[7] Hogg K, Price EM, Hanna CW, et al Prenatal and perinatal environmental influences on the human fetal and placental epigenome. Clin Pharmacol Ther 2012;92:716–26. 10.1038/clpt.2012.141 23047650

[8] Jin B, Li Y, Robertson KD DNA methylation: superior or subordinate in the epigenetic hierarchy? Genes Cancer 2011;2:607–17. 10.1177/1947601910393957 21941617 PMC3174260

[9] Day K, Waite LL, Thalacker-Mercer A, et al Differential DNA methylation with age displays both common and dynamic features across human tissues that are influenced by CpG landscape. Genome Biol 2013;14 10.1186/gb-2013-14-9-r102 PMC4053985 24034465

[10] Heijmans BT, Tobi EW, Stein AD, et al Persistent epigenetic differences associated with prenatal exposure to famine in humans. Proc Natl Acad Sci U S A 2008;105:17046–9. 10.1073/pnas.0806560105 18955703 PMC2579375

[11] Tobi EW, Lumey LH, Talens RP, et al DNA methylation differences after exposure to prenatal famine are common and timing- and sex-specific. Hum Mol Genet 2009;18:4046–53. 10.1093/hmg/ddp353 19656776 PMC2758137

[12] Tobi EW, Goeman JJ, Monajemi R, et al DNA methylation signatures link prenatal famine exposure to growth and metabolism. Nat Commun 2014;5 10.1038/ncomms6592 PMC4246417 25424739

[13] Tobi EW, Slieker RC, Luijk R, et al DNA methylation as a mediator of the association between prenatal adversity and risk factors for metabolic disease in adulthood. Sci Adv 2018;4:eaao4364 10.1126/sciadv.aao4364 29399631 PMC5792223

[14] Joubert BR, Håberg SE, Nilsen RM, et al 450K epigenome-wide scan identifies differential DNA methylation in newborns related to maternal smoking during pregnancy. Environ Health Perspect 2012;120:1425–31. 10.1289/ehp.1205412 22851337 PMC3491949

[15] Park JH, Stoffers DA, Nicholls RD, et al Development of type 2 diabetes following intrauterine growth retardation in rats is associated with progressive epigenetic silencing of Pdx1. J Clin Invest 2008;118:2316–24. 10.1172/JCI33655 18464933 PMC2373422

[16] Crudo A, Petropoulos S, Moisiadis VG, et al Prenatal synthetic glucocorticoid treatment changes DNA methylation states in male organ systems: multigenerational effects. Endocrinology 2012;153:3269–83. 10.1210/en.2011-2160 22564977 PMC3422463

[17] Glier MB, Green TJ, Devlin AM Methyl nutrients, DNA methylation, and cardiovascular disease. Mol Nutr Food Res 2014;58:172–82. 10.1002/mnfr.201200636 23661599

[18] Foran E, Garrity-Park MM, Mureau C, et al Upregulation of DNA methyltransferase-mediated gene silencing, anchorage-independent growth, and migration of colon cancer cells by interleukin-6. Mol Cancer Res 2010;8:471–81. 10.1158/1541-7786.MCR-09-0496 20354000

[19] Ruemmele FM, Garnier-Lengliné H Why are genetics important for nutrition? Lessons from epigenetic research. Ann Nutr Metab 2012;60 Suppl 3(Suppl 3):38–43. 10.1159/000337363 22614817

[20] Slieker RC, Bos SD, Goeman JJ, et al Identification and systematic annotation of tissue-specific differentially methylated regions using the Illumina 450k array. Epigenetics Chromatin 2013;6 10.1186/1756-8935-6-26 PMC3750594 23919675

[21] Seisenberger S, Andrews S, Krueger F, et al The dynamics of genome-wide DNA methylation reprogramming in mouse primordial germ cells. Mol Cell 2012;48:849–62. 10.1016/j.molcel.2012.11.001 23219530 PMC3533687

[22] Seisenberger S, Peat JR, Hore TA, et al Reprogramming DNA methylation in the mammalian life cycle: building and breaking epigenetic barriers. Philos Trans R Soc Lond B Biol Sci 2013;368 10.1098/rstb.2011.0330 PMC3539359 23166394

[23] de Man YA, Hazes JMW, van der Heide H, et al Association of higher rheumatoid arthritis disease activity during pregnancy with lower birth weight: results of a national prospective study. Arthritis Rheum 2009;60:3196–206. 10.1002/art.24914 19877045

[24] Järnerot G, Into-Malmberg MB, Esbjörner E Placental transfer of sulphasalazine and sulphapyridine and some of its metabolites. Scand J Gastroenterol 1981;16:693–7. 10.3109/00365528109182032 6119765

[25] D'Anna-Hernandez KL, Ross RG, Natvig CL, et al Hair cortisol levels as a retrospective marker of hypothalamic-pituitary axis activity throughout pregnancy: comparison to salivary cortisol. Physiol Behav 2011;104:348–53. 10.1016/j.physbeh.2011.02.041 21397617 PMC3118940

[26] Benediktsson R, Calder AA, Edwards CR, et al Placental 11 beta-hydroxysteroid dehydrogenase: a key regulator of fetal glucocorticoid exposure. Clin Endocrinol 1997;46:161–6. 10.1046/j.1365-2265.1997.1230939.x 9135697

[27] Burton PJ, Waddell BJ Dual function of 11beta-hydroxysteroid dehydrogenase in placenta: modulating placental glucocorticoid passage and local steroid action. Biol Reprod 1999;60:234–40. 10.1095/biolreprod60.2.234 9915986

[28] de Steenwinkel FDO, Hokken-Koelega ACS, Hazes JMW, et al The influence of foetal prednisone exposure on the cortisol levels in the offspring. Clin Endocrinol 2014;80:804–10. 10.1111/cen.12388 24350658

[29] de Man YA, Dolhain RJEM, van de Geijn FE, et al Disease activity of rheumatoid arthritis during pregnancy: results from a nationwide prospective study. Arthritis Rheum 2008;59:1241–8. 10.1002/art.24003 18759316

[30] Ince-Askan H, Hazes JMW, Dolhain RJEM Is disease activity in rheumatoid arthritis during pregnancy and after delivery predictive for disease activity in a subsequent pregnancy? J Rheumatol 2016;43:22–5. 10.3899/jrheum.150565 26628599

[31] Ince-Askan H, Hazes JMW, Dolhain RJEM Identifying clinical factors associated with low disease activity and remission of rheumatoid arthritis during pregnancy. Arthritis Care Res. In Press 2017;69:1297–303. 10.1002/acr.23143 27813290

[32] Kooijman MN, Kruithof CJ, van Duijn CM, et al The Generation R Study: design and cohort update 2017. Eur J Epidemiol 2016;31:1243–64. 10.1007/s10654-016-0224-9 28070760 PMC5233749

[33] de Steenwinkel FDO, Dolhain RJEM, Hazes JMW, et al Does prednisone use or disease activity in pregnant women with rheumatoid arthritis influence the body composition of their offspring? Reprod Toxicol 2017;71:118–23. 10.1016/j.reprotox.2017.05.002 28499744

[34] Lehne B, Drong AW, Loh M, et al A coherent approach for analysis of the Illumina HumanMethylation450 BeadChip improves data quality and performance in epigenome-wide association studies. Genome Biol 2015;16 10.1186/s13059-015-0600-x PMC4365767 25853392

[35] Houseman EA, Accomando WP, Koestler DC, et al DNA methylation arrays as surrogate measures of cell mixture distribution. BMC Bioinformatics 2012;13 10.1186/1471-2105-13-86 PMC3532182 22568884

[36] Reinius LE, Acevedo N, Joerink M, et al Differential DNA methylation in purified human blood cells: implications for cell lineage and studies on disease susceptibility. PLoS One 2012;7:e41361 10.1371/journal.pone.0041361 22848472 PMC3405143

[37] Gruzieva O, Xu C-J, Breton CV, et al Epigenome-wide meta-analysis of methylation in children related to prenatal NO2 air pollution exposure. Environ Health Perspect 2017;125:104–10. 10.1289/EHP36 27448387 PMC5226705

[38] Reese SE, Xu C-J, den Dekker HT, et al Epigenome-wide meta-analysis of DNA methylation and childhood asthma. J Allergy Clin Immunol. In Press 2018. doi:10.1016/j.jaci.2018.11.043. [Epub ahead of print: 21 Dec 2018]. PMC6556405 30579849

[39] Bonder MJ, Luijk R, Zhernakova DV, et al Disease variants alter transcription factor levels and methylation of their binding sites. Nat Genet 2017;49:131–8. 27918535 10.1038/ng.3721

[40] Joubert BR, Felix JF, Yousefi P, et al DNA methylation in newborns and maternal smoking in pregnancy: genome-wide Consortium meta-analysis. Am J Hum Genet 2016;98:680–96. 10.1016/j.ajhg.2016.02.019 27040690 PMC4833289

[41] Wang J, Zhao Q, Hastie T Confounder adjustment in multiple hypothesis testing. arXiv:150804178, 2015 Available: https://arxiv.org/abs/1508.04178 10.1214/16-AOS1511 PMC6706069 31439967

[42] van Iterson M, van Zwet EW, Heijmans BT, et al Controlling bias and inflation in epigenome- and transcriptome-wide association studies using the empirical null distribution. Genome Biol 2017;18 10.1186/s13059-016-1131-9 PMC5273857 28129774

[43] Devlin B, Roeder K Genomic control for association studies. Biometrics 1999;55:997–1004. 10.1111/j.0006-341X.1999.00997.x 11315092

[44] Bland JM, Altman DG Multiple significance tests: the Bonferroni method. BMJ 1995;310:170 10.1136/bmj.310.6973.170 7833759 PMC2548561

[45] McLean CY, Bristor D, Hiller M, et al Great improves functional interpretation of cis-regulatory regions. Nat Biotechnol 2010;28:495–501. 10.1038/nbt.1630 20436461 PMC4840234

[46] Jones MJ, Fejes AP, Kobor MS DNA methylation, genotype and gene expression: who is driving and who is along for the ride? Genome Biol 2013;14 10.1186/gb-2013-14-7-126 PMC4054606 23899167

[47] Nakatochi M, Ichihara S, Yamamoto K, et al Epigenome-wide association of myocardial infarction with DNA methylation sites at loci related to cardiovascular disease. Clin Epigenetics 2017;9 10.1186/s13148-017-0353-3 PMC5432989 28515798

[48] Matsuzaka T, Shimano H GLUT12: a second insulin-responsive glucose transporters as an emerging target for type 2 diabetes. J Diabetes Investig 2012;3:130–1. 10.1111/j.2040-1124.2011.00177.x PMC4020729 24843555

[49] Purcell SH, Aerni-Flessner LB, Willcockson AR, et al Improved insulin sensitivity by GLUT12 overexpression in mice. Diabetes 2011;60:1478–82. 10.2337/db11-0033 21441439 PMC3292321

[50] Jiménez-Amilburu V, Jong-Raadsen S, Bakkers J, et al GLUT12 deficiency during early development results in heart failure and a diabetic phenotype in zebrafish. J Endocrinol 2015;224:1–15. 10.1530/JOE-14-0539 25326603

[51] Benjamin EJ, Rice KM, Arking DE, et al Variants in ZFHX3 are associated with atrial fibrillation in individuals of European ancestry. Nat Genet 2009;41:879–81. 10.1038/ng.416 19597492 PMC2761746

[52] Li C, Wang F, Yang Y, et al Significant association of SNP rs2106261 in the ZFHX3 gene with atrial fibrillation in a Chinese Han GeneID population. Hum Genet 2011;129:239–46. 10.1007/s00439-010-0912-6 21107608 PMC5069458

[53] Liu Y, Ni B, Lin Y, et al Genetic polymorphisms in ZFHX3 are associated with atrial fibrillation in a Chinese Han population. PLoS One 2014;9:e101318 10.1371/journal.pone.0101318 24983873 PMC4077770

[54] Kao Y-H, Hsu J-C, Chen Y-C, et al ZFHX3 knockdown increases arrhythmogenesis and dysregulates calcium homeostasis in HL-1 atrial myocytes. Int J Cardiol 2016;210:85–92. 10.1016/j.ijcard.2016.02.091 26930642

[55] Zaw KTT, Sato N, Ikeda S, et al Association of ZFHX3 gene variation with atrial fibrillation, cerebral infarction, and lung thromboembolism: an autopsy study. J Cardiol 2017;70:180–4. 10.1016/j.jjcc.2016.11.005 28007413

[56] Sun S, Zhang W, Chen X, et al The CAA repeat polymorphism in the ZFHX3 gene is associated with risk of coronary heart disease in a Chinese population. Tohoku J Exp Med 2015;235:261–6. 10.1620/tjem.235.261 25797214

[57] Yang S-A Association study between ZFHX3 gene polymorphisms and obesity in Korean population. J Exerc Rehabil 2017;13:491–4. 10.12965/jer.1735080.540 29114518 PMC5667630

[58] Gao S, Li A, Liu F, et al NCOA5 haploinsufficiency results in glucose intolerance and subsequent hepatocellular carcinoma. Cancer Cell 2013;24:725–37. 10.1016/j.ccr.2013.11.005 24332041 PMC3891053

[59] Liu CY, Feng G-S NCOA5, a molecular link between type 2 diabetes and liver cancer. Hepatobiliary Surg Nutr 2014;3:106–8. 10.3978/j.issn.2304-3881.2014.04.02 25019069 PMC4073315

[60] Shtutman M, Baig M, Levina E, et al Tumor-specific silencing of COPZ2 gene encoding coatomer protein complex subunit ζ 2 renders tumor cells dependent on its paralogous gene COPZ1. Proc Natl Acad Sci U S A 2011;108:12449–54. 10.1073/pnas.1103842108 21746916 PMC3145676

[61] Kiang KM-Y, Leung GK-K A review on adducin from functional to pathological mechanisms: future direction in cancer. Biomed Res Int 2018;2018:1–14. 10.1155/2018/3465929 PMC5976920 29862265

[62] Marcun Varda N, Zagradisnik B, Herodez SS, et al Polymorphisms in four candidate genes in young patients with essential hypertension. Acta Paediatr 2006;95:353–8. 10.1080/08035250500434777 16497648

[63] Ramirez GA, Lanzani C, Bozzolo EP, et al Beta-adducin and sodium-calcium exchanger 1 gene variants are associated with systemic lupus erythematosus and lupus nephritis. Rheumatol Int 2015;35:1975–83. 10.1007/s00296-015-3298-x 26045217

[64] Wang J, Duncan D, Shi Z, et al Web-based gene set analysis toolkit (WebGestalt): update 2013. Nucleic Acids Res 2013;41:W77–W83. 10.1093/nar/gkt439 23703215 PMC3692109

