# Supplementary material for: Altered DNA methylation in children born to mothers with rheumatoid arthritis during pregnancy
Source: Ann Rheum Dis. 2019 May 29;78(9):1198–204. doi: 10.1136/annrheumdis-2018-214930 (PMC6788924; doi:10.1136/annrheumdis-2018-214930)
Supplement: Supplementary data [file annrheumdis-2018-214930supp001.pdf]

Supplemental table. The 147 significant CpGs from the linear mixed model: DNA methylation in blood samples from children born to mothers with RA(FEPRA study) compared with children born to mothers from the general population(Generation R Study).

CpG	Nearest gene (+/- bp)	Chr	Base pairs	Location	Beta	Standard error	P-value
cg06642177	SLC2A12(-122529	6	134496341	-	0.027693315	0.002704662	1.32E-24
cg08867893	ZNF365(+221)	10	64134160	-	0.018410514	0.001828632	7.66E-24
cg06778273	TNFRSF18(+4995)	1	1137117	-	0.022857221	0.002306493	3.77E-23
cg07786668	ZFHX3(-10142)	16	73092391	-	0.02586352	0.002626713	7.11E-23
cg20116574	NCOA5(+435)	20	44718168	Promoter	0.018815575	0.002115599	5.91E-19
cg21160472	ATF3(+119)	1	212782112	Promoter	0.013379402	0.00166464	9.18E-16
cg09000178	CBFB(+294)	16	67063319	-	0.014919311	0.001866453	1.31E-15
cg01067849	WRNIP1(-102)	6	2765587	Promoter	0.010168425	0.001336479	2.78E-14
cg14119392	DICER1(-15866)	14	95623926	Promoter	0.009496235	0.001275776	9.81E-14
cg01485645	MLLT6(+303)	17	36862199	Promoter	-0.044105009	0.005979805	1.64E-13
cg10196289	NSMAF(-196)	8	59572185	-	0.013826063	0.001891212	2.66E-13
cg13018448	,CUTA(+600)	6	33385440	Promoter	0.035622707	0.004917127	4.34E-13
cg01402409	FRAS1(-614)	4	78978133	-	0.034187754	0.004758592	6.75E-13
cg17492326	,ANXA13(-31076)	8	124780746	Promoter	0.013530835	0.001894369	9.15E-13
cg06656994	FAM163A(+903)	1	179713176	Enhancer	0.038178019	0.0053507	9.67E-13
cg13390975	BRIX1(+95)	5	34915890	Promoter	0.012727324	0.001788634	1.11E-12
cg17667988	PTBP1(-73)	19	797342	Promoter	-0.033538136	0.004760613	1.86E-12
cg14810343	CXXC5(-127)	5	139028149	-	0.011939828	0.001706185	2.60E-12
cg17662034	RDH10(+706)	8	74207518	-	0.01366994	0.001961263	3.17E-12
cg22247188	H2AFY2(+1271)	10	71813603	-	0.020553561	0.002959855	3.81E-12
cg22998206	-	12	49239429	-	0.031783604	0.004590532	4.40E-12
cg00742472	HEATR5A(-149)	14	31889912	Promoter	-0.036649507	0.005307146	5.00E-12
cg21423973	TYW1B(+121)	7	72298667	Promoter	0.034153776	0.004952153	5.32E-

							12
cg24334803	ZNF672(+281)	1	249132834	Promoter	0.032219477	0.00469291	6.62E-12
cg11014794	CPEB3(+51162)	10	93999688	-	0.020288544	0.002988909	1.14E-11
cg15591678	ZNF365(+216)	10	64134155	-	0.023828478	0.003516927	1.24E-11
cg08700690	RORA(+35122)	15	60884630	Promoter	0.009395729	0.001389406	1.36E-11
cg17218495	SMARCA4(+170)	19	11071743	Promoter	0.010408213	0.001546442	1.69E-11
cg18238491	SKA1(+73)	18	47901440	-	0.006920639	0.0010313	1.94E-11
cg00476896	-	17	60215463	-	-0.029335846	0.004389098	2.33E-11
cg16930947	-	8	88984447	-	-0.049809031	0.007505862	3.22E-11
cg01458961	PPP3CA(-822)	4	102269425	-	0.022397284	0.003379311	3.41E-11
cg22704520	TYW5(+31)	2	200820451	Promoter	0.015040524	0.002278848	4.11E-11
cg25863503	-	6	27799295	-	0.018114832	0.002760384	5.29E-11
cg23792383	KLF5(+171)	13	73633288	Promoter	0.009986606	0.001528091	6.35E-11
cg03847293	-	1	17734508	-	-0.032462167	0.004970659	6.54E-11
cg08561325	AKIP1(+289)	11	8932965	Promoter	0.016285887	0.002494156	6.59E-11
cg12100751	HENMT1(+95)	1	109203672	Promoter	0.012782918	0.001960274	6.98E-11
cg18342832	-	7	99933797	Promoter	0.008837976	0.0013622	8.70E-11
cg21384971	COPZ2(+601)	17	46114574	-	0.029200877	0.004508859	9.40E-11
cg17607973	MEPCE(+179)	7	100027408	Promoter	0.012264221	0.001904559	1.20E-10
cg22512322	PSMD6(+47)	3	64009096	Promoter	0.010619257	0.001666666	1.87E-10
cg17255450	EVC2(-55)	4	5710372	-	0.016872706	0.002655126	2.09E-10
cg16041611	SRF(+785)	6	43139680	-	0.010561056	0.001664015	2.20E-10
cg11877270	SPRED2(+1048)	2	65658583	-	0.014862583	0.002349815	2.53E-10
cg25707994	DNAJB6(0)	7	157129685	Promoter	0.014060725	0.002226484	2.70E-10

cg03654106	-	19	49539527	-	0.026109337	0.004139079	2.83E-10
cg07479988	DHX57(-281)	2	39103277	Promoter	0.023103709	0.003673311	3.18E-10
cg21538902	ADO(+464)	10	64565003	Promoter	0.018717869	0.00297763	3.25E-10
cg19031844	ZNF536(+347411)	19	31210714	Enhancer	0.026178619	0.004168745	3.39E-10
cg06295548	-	4	146296778	-	-0.021299828	0.003393384	3.45E-10
cg06117184	CKAP2L(+70)	2	113522207	Promoter	0.008425305	0.00134451	3.69E-10
cg04910183	CDC73(-75)	1	193090988	Promoter	0.010437417	0.001666679	3.79E-10
cg10181911	RPE(-268)	2	210867059	Promoter	0.035764362	0.005736336	4.53E-10
cg17483482	-	1	117152162	-	-0.036832852	0.005910728	4.62E-10
cg02461956	NCAPD3(+509)	11	134093940	Promoter	0.01486645	0.002389714	4.94E-10
cg19656070	TAX1BP3(+18)	17	3571978	Promoter	0.01069978	0.001727363	5.85E-10
cg07283595	DHX33(+400)	17	5372003	Promoter	0.023494356	0.003797213	6.12E-10
cg25757017	OLR1(+18303)	12	10306462	Promoter, Enhancer	0.021080747	0.00341288	6.54E-10
cg02286335	ZWINT(-11)	10	58121068	Promoter	0.0149277	0.002433753	8.59E-10
cg15248577	,SFMBT2(+3015)	10	7450456	Promoter	0.013766093	0.002252311	9.84E-10
cg14078059	GNS(-21459)	12	65174660	Enhancer	0.016388831	0.0026961	1.21E-09
cg04875007	RPS2(-21)	16	2014871	Promoter	0.012867742	0.002125921	1.42E-09
cg18764107	LBR(+169)	1	225615669	Promoter	0.012219992	0.002018668	1.42E-09
cg04759220	JMY(+660)	5	78532560	-	0.014868421	0.002458298	1.46E-09
cg08522087	ANKH(0)	5	14871910	-	0.007733637	0.00128734	1.88E-09
cg13579901	DCAF6(-280)	1	167905492	Promoter	0.022337576	0.003720867	1.93E-09
cg02750935	PRELID2(-59)	5	145214981	Promoter, Enhancer	0.007716834	0.001285997	1.97E-09
cg05234169	CCDC59(-78)	12	82752252	Promoter	0.016282794	0.002714563	1.99E-09
cg05884522	NOL7(-44)	6	13615538	Promoter	0.01307324	0.00218602	2.23E-

							09
cg06091566	SAMD11(-523)	1	860621	-	0.026435441	0.004423976	2.29E-09
cg24079591	MARK3(-213)	14	103851511	Promoter	0.015991172	0.002680227	2.43E-09
cg11220663	ADD2(+7)	2	70994863	-	0.022700901	0.003806692	2.47E-09
cg12043722	CORO2B(-38066)	15	68870836	-	0.021346312	0.003589317	2.73E-09
cg07262328	CD44(+269)	11	35160709	Promoter	0.013942252	0.00235062	3.01E-09
cg16163324	RAD54L(-458)	1	46712932	Promoter	0.030437342	0.005137256	3.13E-09
cg23222745	-	6	27791912	-	0.016727733	0.002826838	3.27E-09
cg03243965	HSH2D(+9703)	19	16254516	Promoter	0.009874424	0.001670143	3.37E-09
cg06506598	ZFYVE16(+80028)	5	79783889	Promoter	0.024948733	0.004222809	3.46E-09
cg16370446	FAM200B(-43)	4	15683284	Promoter	0.010397718	0.001763573	3.73E-09
cg02547025	LBH(-97)	2	30454275	Promoter	0.008294564	0.001407597	3.80E-09
cg25335190	-	6	27791899	-	0.01905164	0.003241695	4.18E-09
cg22270384	ASNA1(-41)	19	12848240	Promoter	0.02699067	0.004627302	5.45E-09
cg21028463	MFSD11(+124)	17	74733682	Promoter	0.011167499	0.001916108	5.60E-09
cg15698851	TMEM66(+281)	8	29940391	Promoter	0.014332978	0.002463582	5.96E-09
cg15965583	ENSA(+172)	1	150601949	Promoter	0.025400177	0.004366307	5.98E-09
cg22100652	TRIM27(+57387)	6	28834404	Promoter	-0.026777751	0.004614584	6.52E-09
cg08655589	SLC6A6(+46)	3	14444175	Promoter	0.014234875	0.002454671	6.67E-09
cg17834180	IKZF5(+94)	10	124768240	Promoter	0.015030674	0.002593684	6.83E-09
cg10101470	LRRC8D(-870)	1	90286633	Promoter	0.00739195	0.001276564	7.02E-09
cg21597684	HECW2(-42)	2	197457400	-	0.015548919	0.002685279	7.02E-09
cg19273746	B3GAT2(+700)	6	71666063	Promoter	0.023624873	0.004104604	8.63E-09
cg22870667	-	8	521182	Enhancer	-0.027723903	0.004826716	9.26E-09

cg20718350	ASCL1(+819)	12	103352294	-	0.009430784	0.001644405	9.75E-09
cg18289490	SPATA2(+33)	20	48532070	Promoter	0.013736303	0.002396973	1.00E-08
cg20462855	HEY2(-370)	6	126070385	-	0.0072723	0.001269581	1.02E-08
cg20297566	TBC1D23(+267)	3	99979976	Promoter	0.031187463	0.005450513	1.05E-08
cg08709073	RGS7BP(-243)	5	63802184	-	0.018571888	0.003250119	1.10E-08
cg19080138	HERPUD2(+14)	7	35734733	Promoter	0.013942223	0.002443189	1.15E-08
cg26580332	-	8	143919495	-	0.018298842	0.003209963	1.19E-08
cg03713379	SLC12A2(+117789)	5	127537295	-	0.016365348	0.002875992	1.27E-08
cg18120578	E2F7(-24)	12	77459407	Promoter	0.014961422	0.002635655	1.37E-08
cg02994863	PGM1(-29613)	1	64059297	Promoter	0.013618631	0.002400823	1.41E-08
cg15408407	RPS15(+100)	19	1438438	Promoter	0.006751614	0.001191922	1.47E-08
cg21243939	SAMD4A(-1168)	14	55033137	-	0.015308476	0.002707084	1.56E-08
cg04041942	INTS9(-158)	8	28747613	Promoter	0.01474484	0.002608622	1.58E-08
cg12360123	-	10	79984532	Enhancer	-0.043318426	0.007667128	1.61E-08
cg14229247	ANP32B(-373)	9	100745139	Promoter	0.005090587	0.000901141	1.61E-08
cg05944369	HMGB2(-673)	4	174255616	Promoter	0.017054023	0.003024432	1.71E-08
cg04335562	ZNF77(-56)	19	2945000	Promoter	0.023036961	0.004086777	1.73E-08
cg15012981	MAD2L2(+10644)	1	11741009	Promoter	0.005040813	0.000896171	1.86E-08
cg14225021	ITGB6(-69870)	2	161126482	Promoter, Enhancer	0.011800981	0.002102176	1.98E-08
cg09572053	C6orf1(-143622)	6	34360501	Promoter	0.006279856	0.001121122	2.13E-08
cg26646903	TBCCD1(-92)	3	186285208	Promoter	0.011590163	0.002084438	2.69E-08
cg13841783	-	6	32293117	-	-0.030474363	0.00548179	2.71E-08
cg11706469	FGF8(+420)	10	103535362	-	0.027519708	0.004959609	2.88E-08
cg13425677	MAP3K1(+1215)	5	56112090	-	0.014548024	0.0026336	3.31E-

							08
cg15642758	HDGFRP3(-268)	15	83876612	Promoter	0.013003375	0.002354288	3.33E-08
cg06812693	RBPJ(+794)	4	26323246	-	0.020035197	0.003629075	3.38E-08
cg11336323	-	19	41946040	Promoter	-0.03675832	0.00666397	3.47E-08
cg18913103	FBXW7(-1391)	4	153457599	Promoter	0.016671965	0.003022876	3.48E-08
cg10393416	PLK4(+86)	4	128802077	Promoter	0.011204472	0.002035668	3.71E-08
cg20692684	EPC1(-99070)	10	32735158	Promoter	0.013607155	0.002480731	4.13E-08
cg04551440	KATNAL1(-8)	13	30881194	Promoter	0.017295098	0.003153543	4.15E-08
cg04738301	NTN4(-21)	12	96184580	-	0.006624531	0.001210765	4.47E-08
cg01374398	SCAP(+493)	3	47516975	Promoter	0.02248462	0.004109964	4.48E-08
cg05740879	CDKN1C(+185152)	11	2721866	-	-0.027389961	0.005036612	5.38E-08
cg09636406	RECQL5(+110)	17	73663134	Promoter	0.013516309	0.002488052	5.56E-08
cg10391895	ARL2(+112)	11	64781672	Promoter	0.016185438	0.002980622	5.63E-08
cg07361491	GATA6(+96)	18	19749535	-	0.011950742	0.002201116	5.65E-08
cg23055081	TRIM26(-21)	6	30181315	Promoter	0.019228906	0.003543944	5.77E-08
cg15040188	-	19	621137	-	-0.025437993	0.004696691	6.09E-08
cg07226281	MAPK8IP3(+378)	16	1756622	Promoter	0.009645234	0.001783131	6.33E-08
cg17364044	PELI1(+378)	2	64371202	Promoter	0.012546186	0.00232473	6.78E-08
cg12260146	PDCD6IP(+196)	3	33840237	Promoter	0.030045517	0.005570297	6.90E-08
cg24906202	NEO1(+412)	15	73345212	-	0.013867409	0.002573678	7.12E-08
cg06803925	CENPL(+167)	1	173793126	Promoter	0.009030316	0.001677537	7.32E-08
cg16485682	GATA6(+101)	18	19749540	-	0.006185935	0.001149349	7.36E-08
cg23681311	MAPK1(+67)	22	22221878	Promoter	0.008424076	0.001567243	7.65E-08
cg23243902	SIDT2(-113)	11	117049849	-	0.022369089	0.004171023	8.19E-08

cg19906131	PANX1(+447)	11	93862516	-	0.011899087	0.002224471	8.84E-08
cg25668236	ST8SIA4(+2169)	5	100236841	-	0.010861389	0.002033066	9.17E-08
cg21475747	HNRNPAB(-58)	5	177631425	Promoter	0.012719026	0.00238298	9.43E-08
cg26021273	MNX1(-10742)	7	156814112	-	0.020861613	0.003918929	1.02E-07
cg05300158	SETD7(-127)	4	140477727	-	0.008149892	0.00153159	1.03E-07
cg02613964	-	3	44690321	Promoter	-0.019188607	0.003608882	1.05E-07
cg04678743	COPG2(+106)	7	130353515	Promoter	0.011250205	0.00211627	1.06E-07

bp=base pair; Chr= chromosome; SE= standard error

$ Beta represents the difference in DNA methylation at the given CpG site in children born to mothers with RA(FEPRA study) as compared with children born to mothers from the general population(Generation R Study)

Location in promoter, enhancer or unknown(-)
